# Advances in P300 brain–computer interface spellers: toward paradigm design and performance evaluation

**DOI:** 10.3389/fnhum.2022.1077717

**Published:** 2022-12-21

**Authors:** Jiahui Pan, XueNing Chen, Nianming Ban, JiaShao He, Jiayi Chen, Haiyun Huang

**Affiliations:** School of Software, South China Normal University, Guangzhou, China

**Keywords:** electroencephalogram (EEG), brain-computer interface (BCI), P300 speller, P300 paradigm, hybrid BCI

## Abstract

A brain-computer interface (BCI) is a non-muscular communication technology that provides an information exchange channel for our brains and external devices. During the decades, BCI has made noticeable progress and has been applied in many fields. One of the most traditional BCI applications is the BCI speller. This article primarily discusses the progress of research into P300 BCI spellers and reviews four types of P300 spellers: single-modal P300 spellers, P300 spellers based on multiple brain patterns, P300 spellers with multisensory stimuli, and P300 spellers with multiple intelligent techniques. For each type of P300 speller, we further review several representative P300 spellers, including their design principles, paradigms, algorithms, experimental performance, and corresponding advantages. We particularly emphasized the paradigm design ideas, including the overall layout, individual symbol shapes and stimulus forms. Furthermore, several important issues and research guidance for the P300 speller were identified. We hope that this review can assist researchers in learning the new ideas of these novel P300 spellers and enhance their practical application capability.

## Introduction

A brain-computer interface (BCI) is a technology that directly controls external devices by analyzing the electrical signals sent by the nerves of the brain (Wolpaw et al., 2000; Wolpaw, 2007). It was developed to offer patients with motor neuron disease (MND; Hanagasi et al., 2002), including amyotrophic lateral sclerosis (ALS) and locked-in syndrome, a way to communicate with the outside world and regain their social life to a relatively high extent. There are many types of BCI systems related to the detection method of brain activities, including those based on electroencephalography (EEG), near-infrared spectroscopy, magnetoencephalography, and functional magnetic resonance (He and Liu, 2008; Millán and Carmena, 2010), and the most common are EEG-based BCI systems due to their features of noninvasiveness, portability and high signal responses (Wolpaw et al., 1991; De Vos et al., 2014). EEG signals included P300 (Sellers et al., 2006; Kleih and Kübler, 2013; Xu M. et al., 2013), steady-state visual evoked potential (SSVEP; Müller-Putz and Pfurtscheller, 2008; Wu et al., 2008; Jalilpour et al., 2020), and motor imagery (MI; Devlaminck et al., 2011; Park et al., 2012; Kevric and Subasi, 2017). P300 is a positive event related potential (ERP) component that can be generated during an oddball paradigm. In an oddball paradigm, users were presented with a sequence of events that could be divided into two categories (target and non-target), one of which was rarely presented. The P300 is detected 300 ms after the occurrence of a small probability event stimulus (Sutton et al., 1965). SSVEP is a signal induced by presenting a visual stimulus with a specific flickering frequency. P300 and SSVEPs are commonly used EEG signals that do not require training on subjects, and the information transfer rate (ITR) is relatively high. MI is an EEG signal that the subject can emit without external stimulation (Rao, 2013).

At present, BCI is used in many fields, including communication (Kennedy and Bakay, 1998; Hochberg et al., 2006), medical rehabilitation (Rohani and Puthusserypady, 2015), home automation (Corralejo et al., 2014; Park et al., 2015), and game entertainment (Leeb et al., 2013). The BCI speller is one of the most common applications and has been developed for decades. Different types of spellers based on various EEG signals have been studied. The P300-based BCI speller is the most common BCI speller. The earliest BCI speller is a P300-based speller proposed by Farwell and Donchin, which is the basis of many researchers conducted later. Another relatively common type of BCI speller is an SSVEP-based BCI speller. In addition, there are BCI spellers based on MI, and now there are some spellers that combine two EEG signals, such as P300+SSVEP and P300+MI.

Reviews on P300 spelling systems have been conducted to better understand the progress of this field. In Cecotti (2011), Cecotti gave an overview of BCI spellers, including those based on P300, SSVEP, and MI, and the limitations and challenges were pointed out and proposed to help future research tasks. In Philip and George (2020) and Xu et al. (2021), algorithms applied in the P300 system were introduced. Rezeika et al. (2018) gave a systematic introduction of BCI spellers and mainly focused on some successful spellers proposed since 2010. In recent years, to our knowledge, no review has described P300 spellers about their paradigm design and performance. In this article, we will focus on introducing P300 spellers, especially those proposed in recent years, and mention the paradigm design ideas, including the overall layout, individual symbol shapes, and stimulus forms.

**Figure 1:**
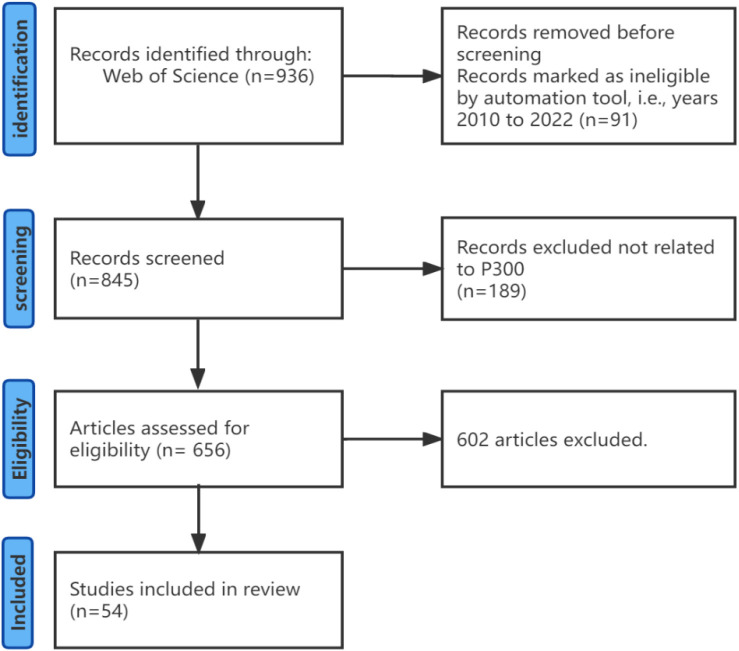
Prisma flow chart.

This review was conducted according to the PRISMA (Preferred Reporting Items for Systematic reviews and Meta-Analyses) guidelines (Moher et al., 2009), as shown in Figure 1. We searched the online database Web of Science and title-abstract keywords (“brain-computer interface” or “BCI”) and (“speller”). First the search was conducted without year constraints and Web of Science showed a total of 936 articles. Later we excluded 91 articles published before 2010 and the remaining 845 were published from 2010 to October 2022. Then 189 articles not related to P300 were excluded and the number of remaining studies was 656 which indicated that nearly 80% of research on BCI spellers was based on P300. Finally we selected 54 of them related to non-invasive BCI and the development of a new graphical user interface.

## Methods

Many efforts have been devoted to the development of the P300 spelling paradigm. On the one hand various parameters of this paradigm have been studied and optimized such as matrix size (Allison and Pineda, 2003), stimulus interval (Sellers et al., 2006), stimulus color (Takano et al., 2009; Zhang et al., 2021), and so on (Xue et al., 2019). On the other hand, researches on developing novel P300 spellers have evolved considerably in the past two decades. In this section, we divide the existing research on P300 spellers into four categories, each of which will highlight some of the representatives, along with their design tenets, paradigms, algorithms, experimental performance, and corresponding advantages.

### Single-modal P300 speller

P300 can be induced in different ways, such as by visual stimuli, auditory stimuli, tactile stimuli, etc. Among them, visual stimuli are the most typically used to evoke the P300, which are also extensively utilized in P300 BCI spellers. The majority of visual spellers are designed based on them. In this subsection, we first reviewed the classic row-column P300 speller and then summarized different structure improvements based on it.

**Figure 2:**
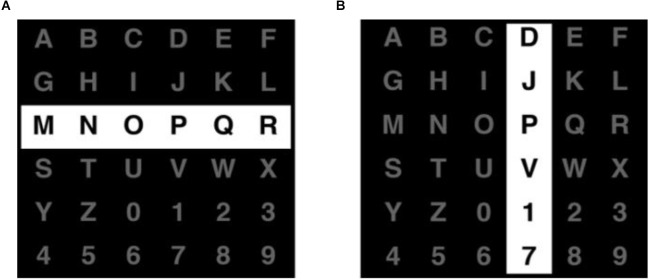
This is an example of the row-column paradigm for the traditional P300 BCI speller. Entire rows **(A)** and columns **(B)** of symbols on a 6×6 grid are flashed randomly.

The first P300 speller was proposed by Farwell and Donchin (1988). The paradigm was a matrix with six rows and columns (row-column paradigm, RCP), as shown in Figure 2. Each row and column were intensified in a random order as the “stimulus event” to induce the P300 potential. At least 12 flashed were needed to cover all items in the matrix. Subjects were instructed to focus on the target letter and count the flashing times of the row and column containing the target to help focus. When the row and column containing the target item flashed, the P300 wave was generated and processed. The system recognized the row and column that elicited the largest P300s. The intersection letter placed at the exact row and column was identified as the target.

The research showed that the typing speed and the maximum accuracy in this study are 12 bits/min and 95%, respectively. This speed was relatively slow compared to the traditional typing methods and seemed to make no sense for healthy individuals, but it was extremely important for those who lacked alternative communication methods. In the development of BCI, the matrix speller played a crucial role since it was the first time that the P300 potential was used to help communication and opened the floodgates to new research of P300.

**Figure 3:**
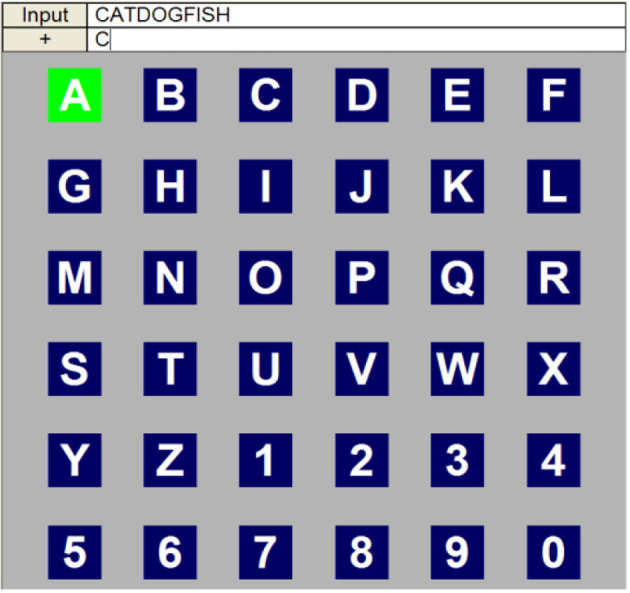
The single display paradigm for the P300 BCI speller. Modified from Pan et al. (2013).

Guan et al. (2004) first proposed the single display (SD) speller. This speller had a 6×6 matrix similar to that of the RCP, and each character was intensified individually. The paradigm is presented in Figure 3. Thirty-six flashes were required to cover all symbols in the matrix, which was three times more than the RCP. In the SD paradigm, the stimulus event that flashed a single character was scarcer than in the RCP, thus eliciting a higher P300 potential. This will also take the subjects more time to catch the target letter in the SD speller. The online experiment showed that the SD speller significantly improved the accuracy. In addition, according to their comparative experiment with RCP, which was conducted based on six subjects, the error rate of the SD paradigm was reduced by up to 80% when 10 trials were used for ensemble average. The SD paradigm was more flexible in that it allowed more kinds of user interface designs, such that the items could spread in different regions rather than gathering in the same square matrix. In addition, the SD paradigm also causes less fatigue, but the typing speed would be lower than that of the RCP.

Fazel-Rezai (2007) showed that in the RCP, subjects tended to have a perceptual error called the “adjacency problem”. Flashes of the rows and columns adjacent to targets seemed to distract the subjects, causing a great number of incorrect detections. To avoid this problem, a region-based speller was proposed by Fazel-Rezai and Abhari (2009). The experimental results in their research indicated that the adjacency problem was reduced by using the region-based paradigm compared with the RCP. Shi et al. (2012) proposed a submatrix-based paradigm, which divided the 6×6 matrix of RCP into several submatrices. Characters in submatrices intensified randomly and individually. The results showed that the submatrix-based paradigm effectively reduced the error caused by the adjacency problem.

Except for the adjacency problem, the “double flash” issue in the RCP was mentioned, which was said that the successive flashes of target might cause wrong detection of P300 (Woldorff, 1993; Martens et al., 2009). To avoid these two problems, the checkerboard paradigm was proposed in Townsend et al. (2010), which was composed of an 8×9 matrix superimposed by an invisible checkerboard. The 36 items placed in the white checkerboard and 36 items in the black checkerboard were reorganized into white and black 6×6 matrices, respectively. The rows in the two matrices were intensified from top to bottom, one by one, followed by their columns from left to right. During the experiment, subjects only saw a standard unchanged matrix with symbols flashing randomly but could not realize the checkerboard. It was proved that the checkerboard paradigm could effectively address the adjacency problem and “double flash” issue.

In subsequent research, many of them tried to modify the paradigm design based on the RCP to improve the performance. In Kaufmann et al. (2011), Kaufmann et al. used the RCP and improved classic character flashing (CF) and novel face flashing (FF) to pixelated face flashing (PFF). CF is the traditional flashing method, while FF is the face flashing method. PFF combined CF and FF to use the intensified rows and columns covered with a familiar face. The experimental results showed that under the PFF mode, subjects’ ERPs increased significantly, which indicated that they reacted faster to the familiar face paradigm and reached higher accuracy than the traditional RCP. Later in Lu et al. (2020), Lu et al. replaced the familiar face with self-faces of subjects and obtained better performance of the spelling system. In Höhne et al. (2011), Höhne et al. proposed an audio speller system with a T9 (text on nine keys) paradigm similar to the nine-key input keyboard used in early cellphones. In Akram et al. (2015), Akram et al. modified the paradigm in Höhne et al. (2011), proposing a visual T9 P300 speller with a dictionary to give suggestions of words to improve typing speed. The experimental results showed that the modified T9 speller significantly reduced the typing time compared to the traditional RCP. Aygün and Kavsaoğlu (2022) expanded the matrix to 7 rows and 7 columns, adding 20 shortcut symbols to select 20 prediction words on the right sight of the screen. Although the 7×7 matrix needed more time to detect a single character, the shortcut elements effectively reduced the time to spell a complete word. According to their comparative experiment, it took only 1.31 min to spell an 8-letter world but 4.53 min for the conventional 6×6 RCP.

The spelling task could also be done in auditory and tactile modality. In van der Waal et al. (2012), van der Waal et al. developed a speller based on tactile stimulation. They used a tactile stimulator and applied it to six of the 10 fingers, each of which represented a character. Subjects selected their desired character by counting the number of tactile stimuli that occurred in the corresponding finger. Chang et al. (2014) presented an auditory speller in Japanese with a two-step input procedure to offer a simple and user-friendly interface. Table 1 gives a summary of the studies mentioned in this subsection.

**Table 1:** Summary of single-modal spellers discussed in this review.

**References**	**Mode**	**Paradigm**	**Classifiers**	**Subjects**	**Acc(%)**	**ITR**
Farwell and Donchin (1988)	P300, visual	RC	SWLDA	4	95%	12 bits/min
Guan et al. (2004)	P300, visual	SD	SVM	6	95%	— —
Fazel-Rezai and Abhari (2009)	P300, visual	RB, two stages	— —	10	— —	— —
Kaufmann et al. (2011)	P300, visual	RC, covering with famous faces	SWLDA	20	Max 100%	— —
Lu et al. (2020)	P300, visual	RC, covering with self-face	BLDA	20	— —	25.8 bits/min
Höhne et al. (2011)	P300, audio	T9	FDA	12	— —	3.4 bits/min
Akram et al. (2015)	P300, visual	T9	RF	10	— —	26.125 s/char
Townsend et al. (2010)	P300, visual	CB	SWLDA	18	92%	23 bits/min
Shi et al. (2012)	P300, visual	SB	SVM	7	— —	— —
van der Waal et al. (2012)	P300, tactile	RB	LDA	12	67%	7.8 bits/min
Chang et al. (2014)	P300, audio	RB	SWLDA	5	— —	— —
Aygün and Kavsaoğlu (2022)	P300, visual	RB	LDA	30	94.56%	— —

RC, row column; SD, single display; RB, region base; T9, text on nine keys; CB, checkerboard; SB, submatrix-based; SWLD, step-wise linear discriminant analysis; SVM, support vector machine; BLDA, Bayesian linear discriminant analysis; FDA, fisher discriminant analysis; R, random forest; Ac, accuracy; ITR, information transfer rate.

There are few P300 spellers based on auditory stimuli or tactile stimuli due to their quite low ITR and accuracy. P300 spellers based on visual stimuli were the most successful. Visual stimuli have an inherent advantage in that they induce higher P300 amplitudes and lower latency and are thus much easier to classify (Aloise et al., 2007). However, some patients who suffer from serious visual impairment have no ability to use visual P300 spellers. In this situation, the non-visual P300 spellers may be an alternative way to communicate with them. Therefore, more attention should be given to spellers based on auditory and tactile stimuli regardless of their relatively poor performance.

### P300 speller based on multiple brain patterns

Many studies have attempted to enhance the performance of the P300 speller system by combining other signals (e.g., P300 and SSVEPs or P300 and MI) with the P300 spellers to improve their performance. For example, the brain patterns induced by the P300 and SSVEP paradigms are different. The utilization of both P300 and SSVEP features from different domains (time domain and frequency domain) is beneficial to construct more efficient BCI systems.

In Yin et al. (2013), Yin et al. first presented hybrid BCI spellers based on the SSVEP and P300, which showed that incorporating other EEG signals into the P300 paradigm was an effective way to improve the performance of speller systems. Later, in Yin et al. (2014), they proposed another hybrid one using two presentation modes: subarea/location and RC mode. In the subarea/location mode, they divided the 6×6 matrix into six groups, and each subgroup flashed at a specific frequency, each of which corresponded to an SSVEP feature. Meanwhile, the symbols in the subgroups flashed with orange crosses, which were used to induce the P300 potential. With the detected SSVEP and P300 signals, the target character can be located. For the RC mode, each column of the matrix flashed at a specific frequency to elicit SSVEPs, and the characters of each row flashed to induce P300. The comparison experiment in the research indicated that the RC mode reached a higher ITR of 53.06 bits/min than the subarea/location mode.

In Santamaría-Vázquez et al. (2019), Santamaría-Vázquez et al. proposed an asynchronous BCI speller based on the P300 and SSVEP. The researchers hold the hypothesis that the peripheral stimuli (i.e., non-target stimuli) of the RCP trigger a weak SSVEP in the user’s EEG. In this article, they put forward two kinds of analysis. The first was to determine how the characteristics of SSVEPs change as a function of stimulus frequency, and the second was to determine the reason for triggering SSVEPs and evaluate their independence from transient ERPs. The conclusion was drawn from the controlled and non-controlled operations. In the control state, the SSVEPs of the EEG signal can be detected. Conversely, SSVEPs should not be found in the noncontrol state. The spelling paradigm was similar to the RCP. The author proposed a novel algorithm called the oddball steady response detection method, which provided a binary output. If the detection was a control state, the system used stepwise linear discriminant analysis (SWLDA) to select a command. In contrast, if the detection was not controlled, no further action was taken. Finally, 60 real controlled observations and 60 synthetic non-control observations of the training set are used to evaluate the performance of oddball steady response detection in offline sessions and online sessions. The results showed that the average accuracy of this speller can reach 95.5%.

Reference (Jalilpour et al., 2020) introduced a hybrid BCI system using rapid serial visual presentation (RSVP) in conjunction with SSVEP. The RSVP (eliciting a P300 response) speller is gaze-independent and space-independent, so it can be used by persons with visual impairment. The proposed paradigm in Jalilpour et al. (2020) divided 27 characters into nine groups, and each group included three characters. The character groups were presented in triple RSVP, and simultaneously, a white square was placed in the center of the screen surrounded by a group of three characters flickering at a fixed frequency. When the subjects looked at each of three different characters, the spatial maps related to the SSVEP potential were different, which was used to identify the target character of the group. Since the P300 and SSVEPs played different roles in this paradigm, researchers used different ways to extract and classify the two signals. In the feature extraction, the authors used discrete wavelet transform and canonical correlation analysis for the P300 and SSVEP, respectively. In addition, they used regularized linear discriminant analysis to classify the P300 potential, and as the SSVEP had a multiclass feature, the researchers used two classifiers, radial basis function (RBF) and support vector machine (SVM), called RBF-SVM classification, to distinguish the correct directions in the SSVEP mode. For the performer of this speller, research showed that it could achieve an accuracy of 93.06% and an ITR of 23.41 bits/min averaged from the data of six subjects. Compared with the single RSVP and triple RSVP spellers in Acqualagna and Blankertz (2013) and Lin et al. (2018), respectively, this speller can reach an accuracy almost as high as the single RSVP and an ITR higher than both. It can be seen that RSVP in conjunction with SSVEP is a recommended way to raise the ITR of the only simple RSVP speller.

In Hwang et al. (2012), a QWERTY (nickname of the traditional keyboard) keyboard layout was first used as the SSVEP speller paradigm by. The QWERTY keyboard layout is that the characters and numbers are arranged in the sequence as the standard keyboard. In Katyal and Singla (2021), Katyal and Singla added the P300 signal into the QWERTY SSVEP speller and achieved a relatively high ITR. In their hybrid speller paradigm, the 36 symbols in the traditional QWERTY SSVEP speller were separated into two sets, one of which included four groups and the other of which included five groups of symbols. Each group had four symbols. It used only five flickering frequencies to induce SSVEPs, while the traditional QWERTY SSVEP speller had 36 different frequencies, which were so close to each other that they reduced the accuracy of the system. The P300 was used to identify the target set, separate 36 symbols and reduce the number of flickering frequencies, so the gap between frequencies could be widened and classification accuracy was improved significantly. The average accuracy and ITR of this hybrid QWERTY speller reached 96.42% and 131 bits/min, respectively. Except for SSVEP, Yu et al. achieved an asynchronous control speller system by combining a MI signal as a switch to turn on/off the speller system (Yu et al., 2016). Table 2 is a summary of the studies mentioned in this subsection.

**Table 2:** Summary of BCI spellers based on SSVEP and P300.

**References**	**Mode**	**Paradigm**	**Classifiers**	**Subjects**	**Acc (%)**	**ITR (bpm)**
Santamaría-Vázquez et al. (2019)	P300+SSVEP, visual	RC	SWLDA	20	95.5	— —
Jalilpour et al. (2020)	P300+SSVEP, visual	triple RSVP	RLDA for P300, RBF-SVM for SSVEP	6	93.06	23.41 bits/min
Yin et al. (2013)	P300+SSVEP, visual	RC	SWLDA for P300, CCA for SSVEP	12	93.85	56.44 bits/min
Yin et al. (2013)	P300+SSVEP, visual	SL RC	SWLDA for P300, CCA for SSVEP	14	— —	44.7 bits/min
						53.06 bits/min
Katyal and Singla (2021)	P300+SSVEP, visual	QWERTY	BLDA for P300, CCA for SSVEP	20	96.42	131 bits/min
Yu et al. (2016)	P300+MI, visual	RC	SWLDA for P300, LDA for MI	11	92.93	41.23 bits/min

SSVEP, steady state visual evoked potential; MI, motor imagery; RC, row column; RSVP, rapid serial visual presentation; SL, subarea/location; SWLDA, stepwise linear discriminant analysis; RLDA, regularized linear discriminant analysis; RBF-SVM, radial basis function and support vector machine; CCA, canonical correlation analysis; BLDA, Bayesian linear discriminant analysis; Acc, accuracy; ITR, information transfer rate.

Ideally, the P300 speller combined with other signals can reach a relatively high ITR compared with the single-modal P300 speller. However, in actual situations, individuals react differently to different kinds of EEG signals. For example, some subjects could produce an intense P300 potential but a weak SSVEP and *vice versa*. Because the spellers depended on multimodal brain signals, if any one of them could not be well detected, the whole spelling system would not work properly. In addition, a speller should not only allow the users to do spelling but also spell at their own pace, so achieving asynchronous control became necessary. One method was to merge MI into speller systems, but MI required long training sessions in subjects, which increased the usage cost of spellers. Future work can attempt to combine other signals (EEG or others) to achieve asynchronous control.

### P300 speller with multisensory stimuli

Researchers have developed P300 BCI based on various kinds of stimuli, including visual P300 BCI (Kaufmann et al., 2011; Li et al., 2015), auditory P300 BCI (Guo et al., 2010; Halder et al., 2010; Xu H. et al., 2013), and tactile P300 BCI (Brouwer and van Erp, 2010), with the intention to be used more universally. The most common P300 BCI is the visual-based BCI since it performs much better than the other types of stimuli, as is the P300 speller. It is difficult to find a P300 speller based on audio-only or tactile-only stimuli. Recently, some researchers have proposed P300 spellers based on multiple senses. In reference Sellers and Donchin (2006), Sellers and Donchin designed a simple paradigm providing three presentation modes, auditory, visual, and audiovisual, and texted it with ALS patients. The result found that it is feasible to use audiovisual stimuli with ALS patients. In reference Belitski et al. (2011), Belitski et al. introduced a matrix audiovisual P300 speller, which numbered every row/column, and each of them intensified with a spoken number. The results showed that the performance of this multisensory speller slightly outperformed both the uni-visual and uni-audio P300 spellers. This paradigm laid the groundwork for later research.

**Figure 4:**
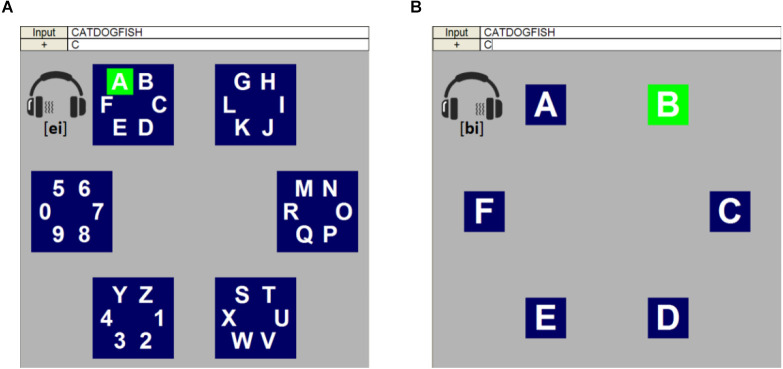
This is an example of a regional audiovisual paradigm for a P300 BCI speller. **(A)** The layout of level 1. **(B)** The layout of level 2, which corresponded to the region placed in the upper left corner of level 1. Modified from Pan et al. (2013).

Lu et al. (2019a) proposed a two-level audiovisual paradigm on the basis of the traditional regional flashing paradigm. As shown in Figure 4, the paradigm included two levels: level 1 was made up of six group areas, each of which included six characters (Figure 4A); level 2 consisted of six single characters spreading from one group area of level 1 (Figure 4B). In level 1, six areas were arranged in three rows and two columns, numbered from top to bottom and from left to right. Each area was intensified randomly and was selected with a broadcast of its serial number from one sight of the earphone (Figure 4A). Similarly, in level 2, six characters were arranged into a 3×2 matrix, randomly intensified, and each was targeted with a broadcasting of its pronunciation (Figure 4B). It should be noted that the sound coming from the side (left/right) of the earphone corresponded to the side of the column (left/right), ensuring spatial and semantic congruence, which can make some areas of the brain more activating, thus helping more accurate classification (Plank et al., 2012).

The feature extraction of the speller is based on temporal and spatial features of the EEG data. For the temporal feature, the researchers chose the time window with differences between the target and non-target stimuli and with evident amplitudes evoked by target stimuli. The *r*^2^ values were used to choose the channels. The calculation method is shown in formula (Wolpaw, 2007). In the process of classification, researchers used Bayesian linear discriminant analysis, an extension of Fisher’s linear discriminant analysis, to classify the data. In the offline experiment, fivefold cross-validation was adopted to calculate the individual accuracy.


(1) $$r^{2}={\left(\frac{\sqrt{N_{1}N_{2}}\left(mean\left(x_{1}\right)-mean\left(x_{2}\right)\right)}{\left(N_{1}+N_{2}\right)std\left(x_{1}\cup x_{2}\right)}\right)}^{2}$$

*N*_1_ is the sample size of the target; *N*_2_ is the sample size of the non-target; *x*_1_ represents the feature vector of the target; *x*_2_ and represents the feature vector of the target.

Researchers compared the paradigm with the unimodal visual speller and found that in some areas, the latency of P3a was significantly shorter and the amplitudes of P3b were higher in the audiovisual paradigm. However, the length of the time to ensure the pronunciation for each character and transition between the two levels might reduce the speed of character spelling.

In Oralhan (2019), Oralhan generated a two-stage audiovisual region-based P300 speller. In the first stage, the author divided the 30 characters into five groups and numbered each of them. The group characters intensified with the speakers playing its serial number. At the second stage, six characters in the detected region at the first stage are displayed separately, flashing with their pronunciation played by the speakers. In the process of signal classification, the researcher used SWLDA, which is a type of Fisher linear discriminant analysis (Krusienski et al., 2006; Hoffmann et al., 2008) that is widely used in P300 spellers due to its higher signal classification accuracy in P300-based BCI applications (Farwell and Donchin, 1988; Krusienski et al., 2006, 2008). In this research, the author compared the proposed speller’s performance with the audio-only and visual-only paradigms and found that the proposed one obtained the highest average accuracy of 90.31% and 78.06% and 54.08% of the visual-only and audio-only paradigms, respectively. In addition, the audiovisual speller reached an average ITR of 6.12 bits/second, which was higher than that of the other two spellers used in the experiments.

The audio stimuli of hybrid BCI speller systems cannot be limited to use as semantic clues. In Lu et al. (2019b), Lu et al. proposed an audiovisual region-based speller, which used smiling faces and audible chuckles corresponding to the smiling faces as visual and audio stimuli, respectively. This cross-modal system proved that the audio stimuli could compensate for the visual stimuli when users got tired of staring at the screen for long periods of time. In Jiang et al. (2019), Jiang et al. proposed an auditory-tactile P300 speller. Although the performance of the visual independent BCI system was worse than those depending on visualization, it was significant for patients with severe visual dysfunction. Table 3 lists the representative P300 spellers based on multisensory stimuli.

**Table 3:** Summary of P300 spellers based on multisensory stimuli.

**References**	**Mode**	**Paradigm**	**Classifiers**	**Subjects**	**Acc (%)**	**ITR (bpm)**
Belitski et al. (2011)	visual+audio, P300	RC	linear	7	— —	— —
Oralhan (2019)	visual+audio, P300	RB, two stages	SWLDA	7	90.31	6.9
Lu et al. (2019a)	visual+audio, P300	RB, two stages	BLDA	18	— —	— —
Lu et al. (2019b)	visual+audio, P300	RB, two stages	BLDA	19	91	43
Jiang et al. (2019)	audio+tactile, P300	RC	BLDA	6	74	— —

RC, row column; RB, region base; SWLDA, step-wise linear discriminant analysis; BLDA, Bayesian linear discriminant analysis; Acc, accuracy; ITR, information transfer rate.

Multisensory stimuli can help support the loss of a single sensory modality and help BCI users concentrate (Belitski et al., 2011). In addition, the subjects of the comparative experiments reported that in multisensory stimulation, the spelling tasks were easier to comprehend than systems based on single stimuli (Oralhan, 2019). One problem was that sometimes multisensory stimuli had a longer duration than the unimodal stimuli. For example, the duration of an audiovisual stimuli should ensure the pronunciation integrity of each character or other voice prompts, which might increase the stimulus onset asynchrony, resulting in loss in ITR. Researchers should consider setting a proper length of stimulus onset asynchrony and weight between classification accuracy and the spelling speed in the design of P300 speller with multisensory.

### P300 speller with multiple intelligent techniques

Recent studies have used 3-D interfaces and tested the functionality of the novel paradigms in P300-based BCI spellers. In Huang et al. (2019), Huang et al. replaced the 2-D interface with a 3-D picture and proved that the virtual reality system was more effective and suitable for usage. In Qu et al. (2018), Qu et al. used 3-D cubes as visual stimuli to evoke P300. Each cube flashed individually with a 3-D motion. The comparison experiment conducted in this research found that the proposed 3-D paradigm induced a higher P300 potential than the traditional 2-D P300 speller. In addition, subjects reported that they felt more comfortable for the use in 3-D paradigm, which could be explained by the attractive and realistic feature of the 3-D visual scene.

**Figure 5:**
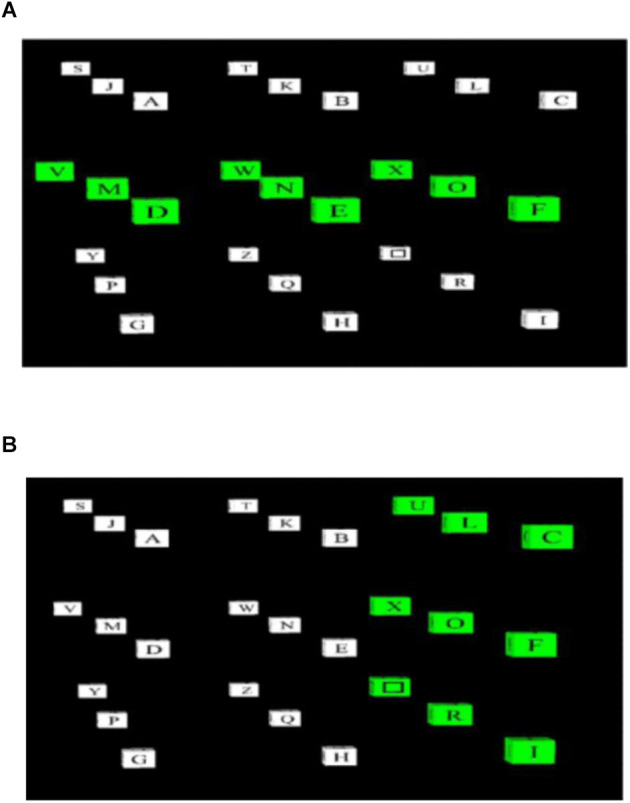
A 3-D virtual paradigm proposed by Noorzadeh et al. (2020). **(A)** Flash on rows. **(B)** Flash on columns. The three depicted target flashes indicate character “F”. Modified from Noorzadeh et al. (2020).

Noorzadeh et al. proposed the 3-D paradigm (Noorzadeh et al., 2020), as shown in Figure 5. The 3-D devices needed to consider how to display 3-D data in a separate image of each eye, so a virtual keyboard was specially set to protect the visual system. It intensified the rows, columns, and depths to cover the symbols, while the classical 2-D paradigm only flashed the rows and columns. The intensified letters would become bigger and the color turned out to be green in the 3-D paradigm to hint the subjects the intensifying row/column/depth. This study compared the classification accuracy and capacity between the 2-D page and the 3-D extended page of the classic P300 speller and verified them through marginal probability, which was based on the experiment of 16 volunteers as the dataset. However, experimental data showed that the classification accuracy of the 3-D interface was lower than that of the classical 2-D paradigm due to the low detection accuracy of the depth planes. This might be because when focusing on the target symbol, the subjects might be distracted by the non-target flashing depth layers. The 3-D paradigm also achieved a lower ITR than the classical 2-D paradigm.

In addition, the authors proposed another flashing approach, which considered each 3-D layer as separate 2-D planes, and all 2-D planes functioned in parallel, called parallel 2-D. The flashing approach of each plane followed the classical row/column paradigm. From the conclusion of the research, the parallel 2-D paradigm reached a similar classification accuracy and ITR as the classical one. The accuracies of the classical 2-D, 3-D, and parallel 2-D methods are 75.16%, 73.25%, and 74.65%, respectively, and the ITRs are 62.4, 58.35, and 62.25 bits/Flash, respectively. In addition, subjects considered the 3-D paradigm to be more comfortable than the classical 2-D paradigm. Seventy-five percent of the subjects preferred the 3-D paradigm for its comfort and ergonomic design in the survey carried out in the experiment.

Inspired by the ideas of the 3-D single flashing paradigm in Qu et al. (2018) and column-only flashing in Ramirez-Quintana et al. (2021), Korkmaz et al. (2022) proposed a 3-D column-only P300 speller in. The columns of the paradigm flashed in a pseudorandom order, and the rows were transposed and intensified also by column. The proposed paradigm not only performed better than the traditional 2-D paradigm but also reduced the workload of the users. In addition, the system used few EEG electrodes, which made it more suitable for practical implementations.

In Du et al. (2019), Du et al. designed three 3-D paradigms in virtual reality (VR) and tried to explore whether higher or similar responses of P300 potentials were induced compared with the 2-D paradigm. They found that stimuli presented with the 3-D effect in VR had better class discriminant than the 2-D paradigm.

Yao et al. (2018) presented an SSVEP speller and achieved good performance. To our knowledge, no complete P300 speller system in VR has been proposed until now, while VR has great potential to evoke higher ERP amplitudes in stimuli presentation. Table 4 lists the representative P300 spellers with multiple intelligent techniques. In future work, research on the P300 speller can focus on presenting the 3-D interface in a VR environment. In addition, researchers can try to address the issue of asynchronous control of these novel P300 spellers in their future work.

**Table 4:** Summary of the P300 speller with multiple intelligent techniques.

**References**	**Mode**	**Paradigm**	**Classifiers**	**Subjects**	**Acc (%)**	**ITR**
Noorzadeh et al. (2020)	P300, visual	RC, 3-D RC, parallel 2-D	BLDA	16	73.25 74.65	58.35 bits/Flash 62.25 bits/Flash
Qu et al. (2018)	P300, visual	RC, 3-D	BLDA	12	94	29.5 bits/min
Korkmaz et al. (2022)	P300, visual	RC, 3-D	ANN	10	91	— —
Huang et al. (2019)	P300, visual	RC, 3-D	BLDA	6	96	42.51 bits/min
Du et al. (2019)	P300, visual	VR, 3-D	LDA	7	— —	— —

RC, row column; 3-D, three dimension; 2-D, two dimension; BLDA, Bayesian linear discriminant analysis; LDA, linear discriminant analysis; ANN, artificial neural network; Acc, accuracy; ITR, information transfer rate.

## Discussion

P300-based BCI spellers are one of the most traditional BCI applications. This article primarily discusses the progress of research into P300 BCI spellers. Several representative P300 spellers were reviewed, including their design principles, paradigms, algorithms, experimental performance, and corresponding advantages. Ever since its concept was first conceived 34 years ago, the P300 has evolved from what was the single-modal speller model designed for only one type of sensory organ into that which is predicated on multiple brain patterns and that featuring multi-sensory stimuli. At this point, researchers are focusing on the angle of multimodal integration to make strides toward optimizing the P300’s performance and user experience. In fact, the most recent iteration highlighting multiple intelligent techniques is the product of relevant optimization that emphasizes the 3D design paradigm and ergonomics.

Compared with other EEG signals such as SSVEP and MI, P300 has its own unique advantage. Many people can produce P300 signals through oddball stimuli, which have been proven to be the most suitable ERP signal. In addition, P300 is well suited for solving problems with many options due to its oddball inducing mechanism and is thus often used in spelling systems. The SSVEP-based paradigm tends to cause visual fatigue in healthy subjects and can even induce epilepsy-like EEG activity in some patient groups, although it seems to be superior to the P300-based paradigm in terms of accuracy and ITR (Allison et al., 2010). MI-based BCI systems need more training time, and many users find it difficult to finish MI tasks without training.

P300 spellers are quite popular due to the relatively stable ERPs and few training requirements among BCI spellers, but inevitably, there are some disadvantages of P300 spellers. First, the ITR of the P300 spellers is relatively low. This is mainly because the P300 spellers were originally designed for patients with severe paralysis. In this regard, the accuracy and reliability of the system is of paramount importance. Additionally, the ITR and accuracy are always trade-offs in the study of BCI applications. Second, the comfort of the P300 spellers needs to be further improved. Most of the existing P300 spellers are designed from a researcher’s point of view and thus lack humane thinking. Third, while using the P300 speller system, subjects are more likely to become visually fatigued with the extension of the usage time, which lowers the detection efficiency of the P300. Then, stimuli of the P300 spellers always require a combination of visual stimuli, and those studies on auditory and tactile stimuli are quite uncommon. As a result, patients with severe paralysis are unable to use the P300 spellers to communicate with others. To improve the above disadvantages, researchers have made many efforts around the system paradigm and algorithm design. For example, Jalilpour et al. (2020) proposed a hybrid paradigm of RSVP spellers and achieved higher classification accuracy and ITR in comparison with previous single-mode spellers. Oralhan (2019) developed a two-stage audiovisual P300 speller based on multisensory stimuli. However, there is still a long way from P300-based BCI to practical application. In addition to the abovementioned directions, researchers should pay more attention to application background and develop more practical systems.

The BCI speller is designed for patients losing their ability to communicate in a traditional way. A visual P300-based speller can be utilized for disables with visual ability. For patients with visual impairment, the multisensory speller may enable them to reclaim their social lives. Audio-visual spellers are suitable for those with deteriorating vision over time (Lu et al., 2019a). Although BCI speller systems have a lower typing rate than traditional typing methods, researchers hope that in the future, BCI spellers will become comparable to traditional tools and use in our daily life. It should be noted that most of the subjects participating in the research on P300 spellers are in good health, which may make the data obtained from the experimental results biased in the use of patients. Specifically, Huang et al. (2021) implemented an asynchronous P300 BCI (variants of the P300 speller) as a communication channel and three patients with disorder of consciousness achieve accuracies significantly higher than chance level. Therefore, to stand on the position of patients, we should invite more end users (e.g., MND patients) to participate in the experiment and consider more from the aspects of appearance, paradigm, algorithm, and application in the research on P300 speller.

Researchers are trying to look for methods to improve the performance of P300 spellers. One strategy is to optimize the design of the interface, which is the part that the users interact directly. This article mainly focuses on recent developments in the paradigm design of P300-based BCI spellers, which aim to provide a better user experience. At the same time, researchers are trying his best to improve the classification algorithms of the spellers. In Aloise et al. (2013), researchers combined a gaze-independent P300 algorithm with an asynchronous algorithm, but the results were not significantly different. Then, in Schettini et al. (2014), the team successfully added a module that can calibrate the parameters of the classifier automatically to the system so it could reach the optimal accuracy. In Bianchi et al. (2019), researchers proposed a method that can dynamically stop the stimulation process to reduce the number of iterations and improve the accuracy of information transmission. Furthermore, one recent study (Gao et al., 2021) provided a subject-independent P300 BCI using an invariant pattern learning method based on a convolutional neural networks and big EEG data that eliminated the calibration procedure and greatly increased the viability of BCIs.

Many studies on the classification algorithm have been carried out to improve the P300 speller performance in term of information transfer rate and accuracy. However, what users directly interact with is the paradigm design of the systems. The evolution of the system from the users is directly impacted by how comfortable and user-friendly it is. Thus, the friendliness, usability, and flexibility are important factors to assess whether people enjoy using the P300 spellers. In fact, questionnaires and other means have been employed to explore subjective factors in many studies (Pan et al., 2013; Li et al., 2019). Furthermore, it is also recommended that researchers pay more attention to the paradigm design of the P300 speller. For example, utilizing other sensory stimuli helps increase the variety of users. Although there are studies utilizing audio-only and tactile-only stimuli (Höhne et al., 2011; van der Waal et al., 2012; Chang et al., 2014), they have poor results with a quietly low ITR. Recent studies have demonstrated that combining visual stimuli with other types of stimuli can result in good performance (Belitski et al., 2011; Lu et al., 2019a, b). However, there are still some patients with vision issues or late-stage ALS; thus, it still makes sense to conduct research on BCI spellers based on audio and tactile stimuli. In addition, we noticed that different paradigms calculate the ITR and accuracy differently, which means that the comparison between them becomes meaningless. In future research, more scientific comparison experiments should be conducted to attain a more trustworthy and objective result.

## Conclusion

Considerable progress has been made in improving the paradigm design of P300-based BCI spellers. The main aim of this article is to gather and describe information on some relatively successful P300-based BCI spellers, especially those published during this decade. Scientists have worked on BCI spellers to achieve higher accuracy and faster typing speed, although there are still many things to do to make spellers more usable and satisfactory. In addition, researchers should pay more attention to the paradigm design since it is the first factor when end-users judge spelling systems. BCI is a commutation system for patients losing their abilities to communicate in a traditional way. In the future, research should prioritize practicality and comfort and propose a more practical and user-friendly P300 speller system. The different systems discussed below may inspire further studies and improvements.

## Author Contributions

JP: conceptualization, supervision, project administration, and funding acquisition. JP, XC, and HH: methodology and validation. XC, NB, and JH: software. XC, HH, and JC: formal analysis and visualization. JP, XC, and NB: investigation and writing—original draft preparation. JP, XC, NB, and HH: writing —review and editing. All authors contributed to the article and approved the submitted version.
